# Elevated pulse pressure and its associations with demographic and clinical parameters in a clinically representative sample of outpatients with psychotic disorders

**DOI:** 10.1192/bjo.2022.52

**Published:** 2022-04-07

**Authors:** Christopher Holmberg, Jarl Torgerson, Andreas Gremyr

**Affiliations:** Department of Psychotic Disorders, Sahlgrenska University Hospital, and Department of Health and Care Sciences, University of Gothenburg, Gothenburg, Sweden; Department of Psychotic Disorders, Sahlgrenska University Hospital, Gothenburg, Sweden; Department of Psychotic Disorders, Sahlgrenska University Hospital, Gothenburg, and Jönköping Academy for Improvement of Health and Welfare, Jönköping University, Jönköping, Sweden

**Keywords:** Psychotic disorders, schizophrenia, outpatient treatment, comorbidity, primary care

## Abstract

Elevated pulse pressure is associated with metabolic and neurocognitive diseases. Preliminary small-scale studies among patients with psychotic disorders have indicated that these patients had an increased pulse pressure compared with controls. However, it is unclear whether and how these associations are manifested among larger heterogenous samples of patients with psychotic disorders. We examined elevated pulse pressure and its associations with demographic and clinical characteristics in a clinically representative sample of outpatients with psychotic disorders (*n* = 1289). In a subsample (*n* = 343), we also examined associations with six domains of functioning. Controlling for age and cardiovascular disease, body mass index (BMI) and employment status independently predicted the odds ratio of having elevated pulse pressure. Elevated pulse pressure was also primarily associated with the physical domains of functioning. Outpatients with psychotic disorders that have high BMI and are unemployed thus seem to be at increased risk for elevated pulse pressure and should therefore be particularly considered for blood pressure screenings.

Pulse pressure, defined as the difference between systolic and diastolic blood pressure, generally increases with age owing to changes in arterial elasticity and reflects physiological ageing of the cardiovascular system.^1^ The European Society of Hypertension has recognised widened pulse pressure as a distinct risk factor that is separate from elevated systolic blood pressure.^2^

Elevated pulse pressure has also been associated with insulin resistance, all-cause mortality among older adults, cardiovascular disease (CVD) and kidney disease.^3^ Elevated pulse pressure can cause blood–brain barrier dysfunction and subsequent adverse neurological changes that may drive or contribute to cognitive impairment not only among older persons.^4^ Elevated pulse pressure dysregulates cerebral endothelial cells, leading to cerebral microvascular damage, along with excessive pulsatile force, which induces breakdown of the blood–brain barrier, resulting in brain cell impairment.^5^

In a small-scale study, patients with psychotic disorders (*n* = 41) were found to have an increased pulse pressure compared with controls.^6^ Preliminary research has indicated that higher pulse pressure could predict the generalised cognitive deficit in psychotic disorders, confirming the role of metabolic abnormalities in the generalised cognitive deficit and suggesting that treatment of hypertension might be a novel adjunctive therapy target for remediating such deficits.^7^

It is thus important to examine pulse pressure in larger clinically representative psychiatric samples. As blood pressure is routinely measured in most psychiatric settings, and pulse pressure is easily calculated, it could enable the prediction of cardiometabolic and cognitive factors. Furthermore, examining associations with demographic and clinical characteristics could help to identify patients at increased risk of elevated pulse pressure, such as those with reduced access to blood pressure screenings due to their socioeconomic status and functional impairments.^8,9^

We therefore aimed to examine pulse pressure and its associations with demographic and clinical characteristics, and with several domains of functioning among outpatients with psychotic disorders.

## Method

The Department of Psychotic Disorders at Sahlgrenska University Hospital in Gothenburg, Sweden, provides tertiary care for individuals with psychotic disorders. We used already-registered patient data, and ethical approval was obtained from the Swedish Ethical Review Authority (#2020–03010).

An observational cross-sectional study design was employed.^10^ Data from annual outpatient check-ups, conducted by the patient's case manager and physician, were used. This included background information such as demographic information, and results from health check-ups (e.g. disease status). The primary criterion was that patients had completed an annual check-up that was recorded in the hospital quality register between 2016 and 2019. During this period, a total of 2179 patients had an annual check-up recorded; of these, 1289 patients had complete blood pressure and body mass index (BMI) measurements recorded within the same 7 day period as the annual check-up (Supplementary file 1 available at https://doi.org/10.1192/bjo.2022.52).

At a few outpatient units, we also had information about patients’ functioning, assessed using the 12 item World Health Organization Disability Assessment Schedule 2.0 (WHODAS-2.0), a self-administered questionnaire.^11^ This is a general assessment instrument that can be used in both patient and general population settings and has been cross-culturally validated both in patients with mental disorders and in those with somatic disorders.^12^ The questionnaire contains two items corresponding to each domain of functioning (cognition, mobility, household, self-care, social and society), and the domains are directly linked to disability concepts according to the International Classification of Functioning, Disability and Health.^12^ The Swedish version has been psychometrically validated in the patient population.^11^ Specifically, the Swedish instrument was validated against patients’ age, clinically diagnosed comorbidities, living conditions, antipsychotic treatment status and psychotic symptom severity.^11^ Of particular interest for this population and the current study, the previous validation study showed that patients who were rated low on psychotic symptom severity generally also self-assessed as having lower functional impairments.^11^ This indicates that the instrument accounts for psychotic symptomology.

Pulse pressure was used both as a continuous (mm/Hg) and a dichotomous variable. The threshold for elevated pulse pressure was set at ≥60 mm/Hg following the 2018 European Society of Cardiology guidelines.^13^ See Supplementary file 2 for all included variables.

Descriptive (e.g. frequencies and dispersions) and inferential statistics were used to examine the data. Both parametric and non-parametric statistical tests were applied depending on the distribution of data (i.e. normally, or non-normally distributed) and the variable type (e.g. interval level or ordinal). Normality was assessed using the Shapiro–Wilk test and complemented by evaluating histograms with normality curves to display the shape and spread of distributions. Thus, associations and differences were assessed using independent samples *t*-tests, χ²-tests, Mann–Whitney U-tests and Spearman's rho. Multiple linear regression analyses were used to assess how much of the variance (measured with adjusted R^2^) the significant independent variables accounted for with pulse pressure as a dependent variable. Finally, binary logistic regression analyses (enter method) were used to identify independent predictors of elevated pulse pressure (≥60 mm/Hg).

SPSS (v. 26, IBM) was used for all statistical analyses. *P*-values were defined with a two-tailed significance level at 0.05.

## Results

A clinically representative sample of 1289 patients was obtained. The mean age was 52.2 (s.d. = 14.3, min–max: 19–92), 592 (46%) were female, the mean systolic blood pressure was 128.1 (s.d. = 16.7, min–max: 86–222), the mean diastolic blood pressure was 80.2 (s.d. = 10.9, min–max: 48–125) and the mean pulse pressure was 47.9 (s.d. = 13.8, min–max: 13–120). Most patients were on antipsychotic medication (91%) and had been diagnosed with a psychotic disorder for more than 10 years (75%).

Of all patients, 257 (20%) had elevated pulse pressure (≥60). Compared with patients with normal pulse pressure, patients with elevated pulse pressure were significantly older (mean age 58 *v*. 51 years, t = −7.9, *P* = <0.001), a higher proportion were diagnosed with psychotic disorder more than 10 years ago (81% *v*. 74%, *X^2^* = 6.3, *P* = 0.012) and a lower proportion of patients had undergone a medical examination within a year (54% *v*. 63%, *X^2^* = 7.5, *P* = 0.006).

Furthermore, patients with elevated pulse pressure had a higher prevalence of diabetes (22% *v*. 13%, *X^2^* = 12.4, *P* = <0.001), chronic obstructive pulmonary disease (COPD) (7% *v*. 2%, *X^2^* = 5.5, *P* = 0.019), CVD (25% *v*. 12%, *X^2^* = 26.9, *P* = <0.001) and history of cancer (5% *v*. 2%, *X^2^* = 5.7, *P* = 0.017).

Using Spearman's rho, statistically significant associations were found between pulse pressure and the following variables: age (*r* = 0.240, *P* = <0.001), female sex (*r* = 0.097, *P* = 0.001), BMI (*r* = 0.074, *P* = 0.008), CVD (*r* = 0.138, *P* = <0.001), diabetes (*r* = 0.094, *P* = 0.001), COPD (*r* = 0.061, *P* = 0.030), having been born in Sweden (*r* = −0.065, *P* = 0.022), being employed (*r* = −0.091, *P* = 0.002) and mobility (*r* = 0.138, *P* = 0.011).

When controlling for age and CVD, binary logistic regression analysis revealed that BMI (odds ratio [OR] = 1.026, *P* = 0.036) and being employed (OR = 0.617, *P* = 0.003) were independently associated with elevated pulse pressure (Table 1). The unique variance explained by BMI and being employed as predictor variables on pulse pressure was 11% (adjusted R^2^ = 0.11) and 16% (adjusted R^2^ = 0.162), respectively.

**Table 1:** Binary logistic regression with elevated pulse pressure (PP ≥ 60) as dependent variable

	95% CI for EXP(B)						
Predictors for elevated PP	Beta	Wald	d.f.	*P*-value	Exp(B)	Lower	Upper
BMI	0.026	4.403	1	0.036	1.026	1.002	1.052
Employed	−0.483	8.967	1	0.003	0.617	0.449	0.846
Constant	−3.303	24.549	1	0.000	0.037		

BMI, body mass index. Hosmer–Lemeshow test: χ^2^ = 2.182, *P* = 0.975.

The 343 patients with recorded WHODAS-2.0 were similar to patients without such recordings in terms of their age (mean 51.7 *v*. 52.4, t = −0.779, *P* = 0.436) and their sex composition (*X^2^* = 0.037, *P* = 0.847).

Patients with elevated pulse pressure tended to have higher mean scores (more functional impairment) in all dimensions of functioning. However, only the dimensions of household (*P* = 0.043) and mobility (*P* = 0.001) were statistically significant, as was the WHODAS-2.0 sum score (*P* = 0.008) (Table 2).

**Table 2:** Differences in dimensions of functioning between patients with normal (<60) and elevated (≥60) pulse pressure (PP). Numbers represent mean (s.d.) and interquartile range

	PP < 60 (*n* = 271)	PP ≥ 60 (*n* = 72)	*P*-value^a^
Dimensions of functioning			
Cognition	1.71 (0.9), 1.0	1.91 (1.0), 1.5	0.171
Household	1.95 (1.0), 1.5	2.24 (1.1), 2.0	0.043*
Mobility	1.68 (1.0), 1.0	2.28 (1.4), 2.5	0.001**
Self-care	1.23 (0.6), 0.0	1.45 (0.9), 0.5	0.056
Social	1.79 (0.9), 1.5	1.80 (0.8), 1.5	0.533
Society	2.27 (1.1), 1.5	2.47 (1.1), 1.88	0.128
WHODAS-2.0 sum score	21.25 (8.5), 10.0	24.29 (9.4), 14.0	0.008**

Cognition, understanding and communicating; Household, life activities; Mobility, getting around; Self-care, maintaining personal hygiene and dressing independently; Social, getting along with others; Society: participation in society.

a. Mann–Whitney U-test.

*P < 0.05, **P < 0.01.

## Discussion

Similar to previous research in patients without psychiatric disorders, we found that elevated pulse pressure was related to patients’ physical health status indicators such as BMI and presence of diabetes, CVD and COPD.^3^ Elevated pulse pressure was particularly related to the physical dimensions of functioning such as mobility and ability to perform household chores. Unlike previous research, we did not find any statistically significant associations between elevated pulse pressure and cognitive impairments.^7^

When controlling for age and CVD, we found that patients that were employed had a significantly lower OR for elevated pulse pressure (roughly a 40% reduction). This is consistent with research using cohorts from the general population, which also found that employment status was a significant predictor of elevated pulse pressure.^9^ Interestingly, in our study, other aspects of socioeconomic status such as the patients’ education level and living situation were not significantly associated with pulse pressure. The clear association with elevated pulse pressure and being employed might therefore relate to functioning, as increased impairment in physical dimensions of functioning (e.g. mobility) and the sum disability score were associated with elevated pulse pressure, which in turn might explain the patient's ability to work. Another potential explanation for this association might be patients’ income, which we unfortunately did not have information about. Patients in employment tend to have better financial situations, and this might influence their opportunity to access treatment for hypertension and other medical conditions, as observed in previous studies.^8,14^

Blood pressure is regularly measured in psychiatric settings, and pulse pressure is easily calculated. It is therefore worth considering using pulse pressure more actively as a prognostic marker in clinical practice.^15^ These findings suggest that psychotic outpatients with high BMI and patients that are unemployed are the most at risk of elevated pulse pressure, which needs to be considered in clinical practice. A limitation of the current study was its cross-sectional design. Cross-sectional designs have inherent predictive constraints, because the temporal link between exposure and outcome cannot be established as both are examined at the same time. Thus, it is not possible to determine a true cause and effect relationship. Future studies should therefore consider using longitudinal designs. This would allow researchers to examine the predictive validity of pulse pressure more robustly in the identification of health status and functioning in patients with psychotic disorders. Another limitation of the current study was the potential for selection effects to influence the representativeness of the study sample. Of the 2179 patients that had an annual health check-up recorded, 1289 (59%) had complete blood pressure and BMI recordings obtained within the same 7 day period. This could mean that patients with more severe mental or physical disease or disability were not accurately represented in the sample. However, the sample was heterogenous in terms of demographic (e.g. wide age distribution) and clinical (e.g. wide BMI distribution) parameters, which reflects the clinical patient population.

## Data Availability

Study data are not available for sharing owing to ethical approval requirements. Researchers interested in collaboration should contact the corresponding author.

## Supplementary material

For supplementary material accompanying this paper visit http://doi.org/10.1192/bjo.2022.52. click here to view supplementary material
