# Supplementary material for: Elevated pulse pressure and its associations with demographic and clinical parameters in a clinically representative sample of outpatients with psychotic disorders
Source: BJPsych Open. 2022 Apr 7;8(3):e79. doi: 10.1192/bjo.2022.52 (PMC9059612; doi:10.1192/bjo.2022.52)
Supplement: Supplementary file 1 [file S2056472422000527sup001.docx]

**Supplementary file 1.** Sampling procedure.

Patients with annual check-ups, recorded between 2016-2019

(n=2176)

Patients with annual check-up, weight/height, and blood pressure recorded within a 7-day period

(n=1289)

Patients excluded since they ***a)*** did not have the measures recorded within the same 7 days of the annual check-up, or ***b*)** incomplete or apparent incorrect values:

- Weight and height (n=512)
- Systolic and diastolic blood pressure (n=375)

(n=887)

**Supplementary file 2.** List of variables.

Patient background information included: Age (in years), sex (1=female), duration of psychosis diagnosis (1=2009 or earlier), antipsychotic medication (1=yes), education (1=not completed senior high school), living condition (1=independently), employment status (1=employed), systolic and diastolic blood pressure (mm/Hg), BMI (kg/m^2^), cardiovascular disease (CVD, yes/no), diabetes mellitus (yes/no), thyroid disease (1=yes), smoking (1=yes), birthplace (1=Sweden), conducted health interview within the last year (1=yes), and conducted medical examination within the last year (1=yes).
